# Scientific News

**Published:** 1880-07

**Authors:** 


### SCIENTIFIC NEWS.

Mr. Alfred Taylor, in a paper read to the Anthropological Institute, holds that the lower animals have no abstract ideas, and, therefore, all they can know must be derived from objects. Their reproduction of specific form and decoration seems to prove that they possess a mental power of appreciating the niceties of form and color in a very high degree. The forms and decorations of organized beings seem to be regulated by laws which the author provisionally called *emphasis* and *symmetry*. Emphasis was defined as the marking out by form or decoration of the important parts or organs. The law of emphasis, as applied to human work, was illustrated by the structure of a Greek temple, in which all the parts have their functions expressed or emphasized by ornament. It is a remarkable fact, and one that can scarcely be accidental, that just as animals fall naturally into two great classes, the *vertebrata* and *invertebrata*, so the emphasized functional decorations group themselves into two classes, and these two classes are identical with the vertebrata and the invertebrata.

In the vertebrata the emphasized ornamentation is what we may call axial, being the outward expression of the central axis or vertebral column with its appendages; and in the invertebrata the decoration tends to follow the outline of the animal, and so develops borders. It has always excited wonder that the child—a separate individual—should inherit and reproduce the characters of its parents, and, indeed, of its ancestors; but if we remember that the great law of all living matter is that the child is *not* a separate individual, but a part of the living body of the parent, up to a certain date, when it assumes a separate existence, then we can comprehend how living beings inherit ancestral characters, for they are parts of one continuous series in which not a single break has existed or can ever take place. Just as the wave form over a pebble in a stream remains constant, though the particles of water which compose it are ever changing, so the wave-form of life which is heredity remains constant though the bodies which exhibit it are continually changing.—*Stoddart's Review.*

---

M. M. Zweifel and Moustier, two clerks in the employment of M. Verminck's commercial house at Sierra Leone, have reported that their journey to discover the source of the Niger is successful. The Kong mountains were crossed and the main source of the Niger "was found on the frontier of Kirsi and Koranka; in short, near the place indicated on Major Laing's map." Major Laing traveled in that region in 1822.

---

In the Grand Duchy of Nassau, at Leck, during a thunderstorm, recently, it is reported that an electric discharge fell into a pond containing fish, killing them all. They arose to the surface presenting the appearance of having been boiled.

SOME remarkable transformations in the character of the Algerian Sahara have been effected by irrigation. Under its operation a soil has been constituted, in which the inter-tropical plants grow with great vigor. A cultivator in Quargla received several medals at the Parisian Exposition for plants which he had raised on a soil thus prepared. The stories that have been told of the productiveness of the Sahara tax the imagination. Fertility is not limited to any one point. It is exhibited wherever water has been brought to the surface of the soil.

Most of the Saharan valleys and the beds of the subterranean streams have water in abundance, and only a small effort is needed to bring it to the surface. Sahara is not all a desert, but contains many considerable tracts which are already fit for cultivation.

The success which has attended the efforts, so far made to introduce tillage, renders it nearly certain that a like reward may be gained from similar applications of labor in other parts. Henceforth it will be safe to say that the transformation of the Sahara is only a question of time—labor, artesian wells, means of communication, and security.—*Popular Science Monthly.*

---

THE habit of excessive tea-drinking is a wide-spread and deep-rooted evil, the devotees generally being women. Professional tea-tasters, however, furnish the best examples of its noxious effects upon the system. Amongst other disturbances produced by the immoderate use of the plant, Dr. Morton, of New York, enumerates "head-ache, vertigo, heat and flushings of the body, ringing in the ears, mental dullness and confusion, tremulousness, 'nervousness,' sleeplessness, apprehension of evil, exhaustion of mind and body, with disinclination to mental and physical exertion, increased and irregular action of the heart, increased respiration."

---

RUMOR announces the discovery of gold in the district of Chandiere River, Canada; it is proposed to make a scientific survey to ascertain the truth.

---

AS a result of measurement, three hundred Japanese give an average height of five feet, three inches.

INVESTIGATIONS are being made by several British societies to ascertain what degree of protection is afforded by lightning conductors and the preparation of a code of rules for the erection of rods. To make observations accurate and methodical, information is wanted on the following points: "Full details of accidents by lightning, stating especially whether the building struck had a conductor or not. If there was a conductor, state its dimensions, construction, mode of attachment to the building; whether its top was pointed, distance of its upper terminal from the place struck, nature and extent of the connection between the conductor and the earth, and whether the earth was dry or moist; whether the conductor was itself injured, and whether the conductor or the point struck was the most salient object in the vicinity. Information is also desired, either verbally or by sketches, as to the position of metal spouting and lead roofing relatively to the point struck, and to the conductor. Details of the thickest piece of metal melted by a flash of lightning are much needed. Unimpeachable evidence of the failure of conductors is much desired, as such failures would be extremely instructive."

---

SURGEON J. H. Kidder, U. S. N., has proved by a series of investigations that fish are somewhat warmer than the water they live in. The temperature of the dog fish was found 12° higher than that of the water. The degree of heat was determined by opening the fish immediately upon removal from the water and inserting the bulb of the thermometer in the cavity of the heart or bronchial artery, the bloodvessel connecting the gills and heart.

---

MOUNT ETNA is once more quiet; its summit covered with snow. An exploration of the crater is to be made, to note the changes made during the recent eruptions.

---

IN the death of Prof. David Thompson, of Aberdeen University, where he taught natural philosophy for thirty-five years, Science loses another of her hard workers.

---

MR. GRAHAM BELL won the Volta Prize of 50,000 francs.

